# Genetic and genomic stability across lymphoblastoid cell line expansions

**DOI:** 10.1186/s13104-018-3664-3

**Published:** 2018-08-03

**Authors:** Laura B. Scheinfeldt, Kelly Hodges, Jonathan Pevsner, Dorit Berlin, Nahid Turan, Norman P. Gerry

**Affiliations:** 1 Coriell Institute for Medical Research, 403 Haddon Ave, Camden, NJ 08003 USA; 2 Kennedy Krieger Institute, 707 N. Broadway, Baltimore, MD 21205 USA; 3 Advanced BioMedical Laboratories, 1605 Industrial Hwy, Cinnaminson, NJ 08007 USA

**Keywords:** Lymphoblastoid cell line, Single nucleotide polymorphism, Structural variation, Genomic, Stability, Copy number variant

## Abstract

**Objective:**

Lymphoblastoid cell lines are widely used in genetic and genomic studies. Previous work has characterized variant stability in transformed culture and across culture passages. Our objective was to extend this work to evaluate single nucleotide polymorphism and structural variation across cell line expansions, which are commonly used in biorepository distribution. Our study used DNA and cell lines sampled from six research participants. We assayed genome-wide genetic variants and inferred structural variants for DNA extracted from blood, from transformed cell cultures, and from three generations of expansions.

**Results:**

Single nucleotide variation was stable between DNA and expanded cell lines (ranging from 99.90 to 99.98% concordance). Structural variation was less consistent across expansions (median 33% concordance) with a noticeable decrease in later expansions. In summary, we demonstrate consistency between SNPs assayed from whole blood DNA and LCL DNA; however, more caution should be taken in using LCL DNA to study structural variation.

**Electronic supplementary material:**

The online version of this article (10.1186/s13104-018-3664-3) contains supplementary material, which is available to authorized users.

## Introduction

Lymphoblastoid cell lines (LCLs) are a valuable resource to the genetics and genomics communities primarily due to their ability to provide a renewable source of DNA. Other common applications include studies of gene expression and gene regulation [1, 2], drug metabolism [3], immunology [4], cell biology [4], and in the development of iPSCs [5].

Previous work has evaluated the stability of single nucleotide polymorphism (SNP) and copy number variants (CNVs) in cultures [6–9]. Shirley et al. [9] compared SNPs and CNVs between DNA extracted from blood and DNA extracted from Epstein Barr Virus transformed LCLs in a sample of 6 research subjects. Oh et al. [10] compared SNPs between initial and later passage (up to 180) LCLs in a sample of cultures generated from 20 research subjects. Both studies consistently found high SNP concordance (ranging from 98.8 to 99.9%) across technical replicates, biological replicates, and between DNA from blood and transformed cell cultures. CNV concordance was notably lower (median 56%) in results reported by Shirley et al. [9], and Oh et al. [10] reported several regions that were vulnerable to loss of heterozygosity when the number of passages was ≥ 50.

**Fig. 1:**
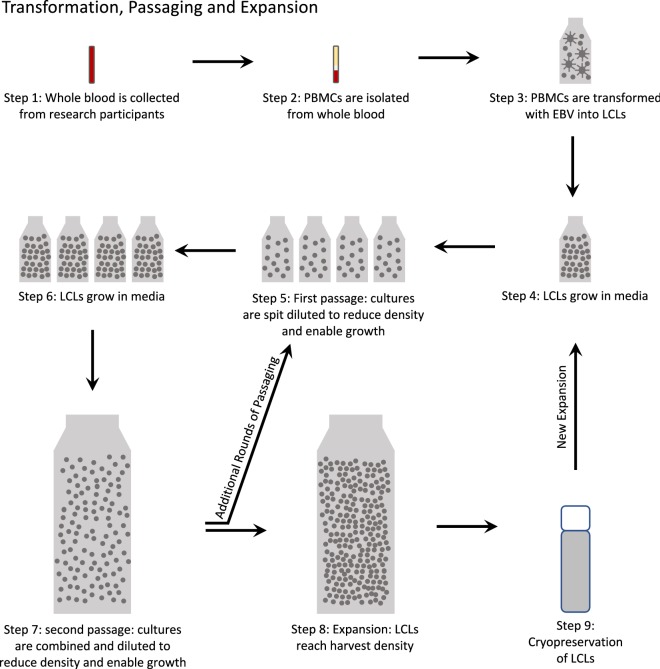
Diagram of cell transformation, passaging and expansion. Whole blood is collected from research participants, and peripheral blood mononuclear cells are isolated and transformed into lymphoblastoid cell lines (LCLs) with Epstein Barr Virus. LCLs are grown in medium as growth plateaus due to high cell density and prior to over-saturation. Passaging (splitting the culture into diluted sub-cultures) enables LCLs to continue to grow over time. To reach expansion volumes, sub-cultured cells are combined into a larger container and diluted with medium to enable further growth (second passage). When the culture again reaches high cell density at high volume the expanded cells are harvested for cryopreservation and made available for DNA or LCL distribution

While cell culture passaging involves diluting cells from culture to reduce cell density and enable ongoing culture growth, cell culture expansion involves the growth of a much larger populations of cells in culture to a defined endpoint to allow for long term storage or distribution. As diagramed in Fig. 1, transformed LCLs are grown in medium until growth plateaus due to high cell density, but prior to over-saturation. Passaging, the splitting of the culture into diluted sub-cultures, enables LCLs to continue to grow over time. In the case of expansion, transformed LCLs are typically passaged prior to being grown to the desired cell counts needed for distribution banks. To date, we are not aware of any published work that evaluates SNP or CNV stability after LCL expansion. Given the common use of biorepository DNA in genetic and genomic studies of human variation, and the regular use of cell culture expansion in biorepository operations, we have extended the evaluation of SNP and CNV stability across LCL expansions.

## Main text

### Methods

#### Subjects

Biospecimens were collected from six research subjects. Written informed consent was obtained, and the study was conducted with approval from the Coriell Institute for Medical Research Institutional Review Board, as previously described in Shirley et al. [9]. Six tubes of whole blood were collected from each individual, and peripheral blood mononuclear cells (PBMCs) were isolated from these samples.

#### Cell culture

For each research participant, whole blood from one tube was used for DNA extraction, and the remaining five tubes of blood were used to establish five separate LCLs. As outlined in Fig. 1, PBMCs were independently isolated from each tube of whole blood (Fig. 1, Steps 1–2) followed by transformation with Epstein Barr Virus (Fig. 1, Step 3; as previously described in Shirley et al. [9]). Once these initial LCLs were established, foundation stocks were cryopreserved for DNA extraction as well as for future passaging and expansion in order to generate large distribution banks of cells. The expansion process began with growing the LCLs until the culture density reached near saturation (Fig. 1, Step 4). To enable further growth, each LCL culture was split into four diluted flasks, and this step is considered the first passage (Fig. 1, Step 5). When the growth in these flasks plateaued again (Fig. 1, Step 6), the cultures were combined and diluted in a larger roller bottle to enable further growth, creating the second passage (Fig. 1, Step 7). Once each culture reached harvest density, it was considered the first expansion (Fig. 1, Step 8). Cryopreserved LCLs (Fig. 1, Step 9) were then put through the process again beginning with growing expanded LCLs in media (Fig. 1, Step 4) for additional expansion generations. Between the second and third expansion, additional passaging took place.

In total, 29 LCLs were successfully established (Additional file 2: Table S1; [9]) and sequentially expanded three times. Creation of the additional expansions followed the culturing procedure outlined above starting with LCLs from the first expansion to create expansion 2, and LCLs from expansion 2 to create expansion 3. Between the first and second expansion each LCL was passaged 2 times and between the second and third expansion each LCL was passaged 10 times. In addition, technical replicates of the original freeze from two LCLs were processed, and technical replicates of the first expansion from four LCLs were constructed (Additional file 1: Figure S1, Additional file 2: Table S1).

LCLs used in the current study are listed individually in Additional file 2: Table S1, and these samples are available as part of the NIGMS Human Genetic Cell Repository at the Coriell Institute for Medical Research (https://catalog.coriell.org/1/NIGMS).

#### Genomic data collection and analysis

One hundred and twenty-nine DNA samples, which included 7 technical replicates were processed for analysis on the Affymetrix Human SNP Array 6.0 (Affymetrix 6.0; Affymetrix, Inc. Santa Clara, CA). CEL files were input into Affy Power Tools (APT 1.16; https://www.thermofisher.com/us/en/home/life-science/microarray-analysis/microarray-analysis-partners-programs/affymetrix-developers-network/affymetrix-power-tools.html) to generate CHP files for each sample, and B allele frequencies and log R ratios were calculated for each sample and for each SNP. PennCNV was used to call CNVs from the B allele frequencies, log R ratios, and signal files for each sample set of autosomal chromosomes using default settings [11]. We retained CNV calls containing at least 25 SNPs for further analysis. In cases where individual PennCNV calls shared overlapping regions, we consolidated them into a single CNV region. We additionally incorporated the four immunoglobulin regions as defined in the PennCNV documentation as an additional results filter [11]; although we used the UCSC Genome Browser Liftover tool to convert the coordinates to B37 [12]. In addition, there was one set of duplicated CNVs on chromosome 17 in the third expansion of sample GM22644 that were treated as a single region, and another set of duplicated CNVs on chromosome 11 in the original GM22679 culture and the first two expansions of this sample that were treated as a single region. Plink 1.90 (http://www.cog-genomics.org/plink/1.9/) [13] was used to calculate SNP concordance (the proportion of shared SNPs that are identical by state), and a custom R script was developed to calculate CNV concordance (the proportion of shared CNVs between samples; available upon request).

### Results and discussion

#### SNP stability

**Fig. 2:**
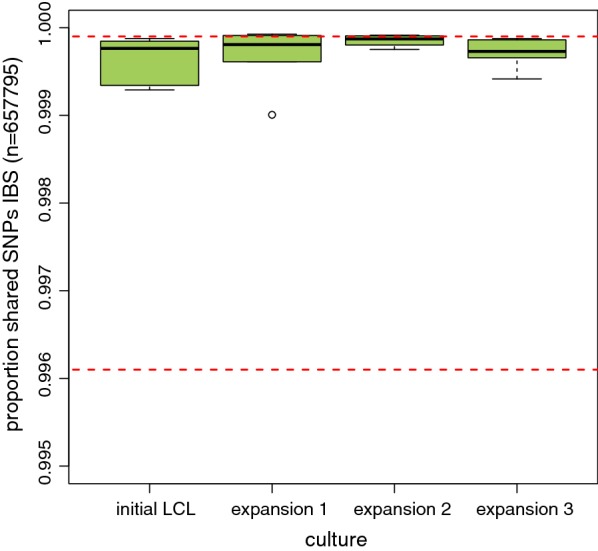
SNP concordance across cell culture expansions. This figure displays boxplots of the distribution of pairwise concordance between DNA (from blood) and cell culture for each individual subject. Concordance for the initial transformed cell culture and three expansions cultures are shown along the X-axis. The Y-axis displays the percentage of SNPs that were estimated to be shared using the identity by state method [13]. Red dash lines indicate the minimum and maximum SNP concordance across technical replicates

SNP stability was generally consistent across expansions. As displayed in Fig. 2, SNP concordance ranges from 99.90 to 99.98% across expansions, and this range was well within the range of SNP concordances observed across technical replicates (0.9961–1.0000). These SNP concordances were also within the reported technical reproducibility of the Affymetrix SNP 6.0 Array (99.9%) [9] and the average concordance rate of SNP 6.0 data analyzed with the Affymetrix Birdseed algorithm (99.8%) [14].

#### CNV stability

**Fig. 3:**
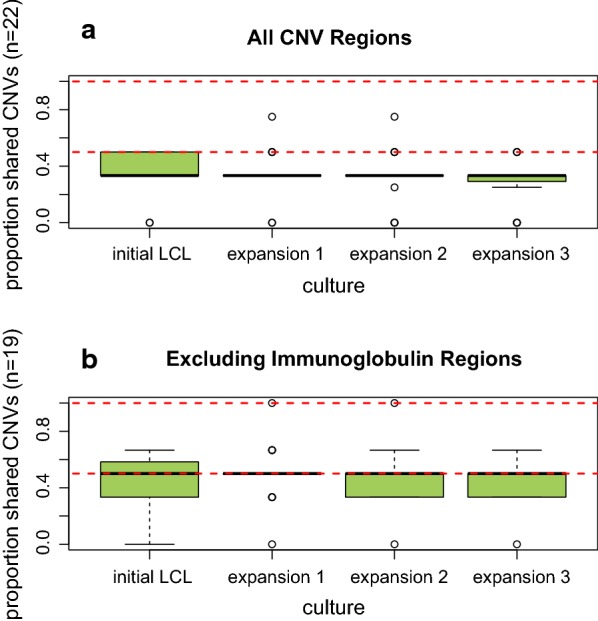
CNV concordance across cell culture expansions. This figure displays boxplots of the distribution of pairwise shared CNV regions between DNA (from blood) and cell culture for each individual subject. Shared CNV regions for the initial transformed cell culture and three expansions cultures are shown along the X-axis. The Y-axis displays the percentage of CNV regions that were estimated to be shared using PennCNV calls [11]. Red dash lines indicate the minimum and maximum number of shared CNVs across technical replicates. **a** Displays results for all CNV regions, and **b** displays results that exclude the three immunoglobulin regions identified in the samples

In total, we identified 22 unique CNV regions across all 129 samples (Additional file 3: Table S2); however, 3 of these were immunoglobulin (IG) regions that have been previously implicated in cell culture artifacts [11]. CNV regions ranged from approximately 45 kb to 50 Mb in size (average region size = 174 kb; median region size = 199 kb). When including IG regions, CNV concordance across expansions was lower (median 0.33) than was previously reported for first passage LCLs (median 0.56 [9]). Concordance increased noticeably with the exclusion of IG regions (median 0.50). Figure 3 displays CNV region sharing across each expansion. Unlike SNP concordance, CNV concordance was almost completely outside of the range of CNV concordance across technical replicates. Given the relatively small subject sample size (n = 6), and modest number of CNV calls (n = 22), these concordance estimates cannot fully address the question of CNV stability across LCL expansion. However, these preliminary results suggest that CNV stability may decrease over expansion generations.

### Conclusions

The combined evidence indicates that SNPs are generally stable after EBV transformation [9], across early (< 50) passages [10], and across three culture expansions (current study results). Some genomic regions are vulnerable to structural instability in the transformation process [9], and other genomic regions are vulnerable to loss of heterozygosity in later passages (≥ 50) [10]; our analysis of structural variation suggests a potential decrease in CNV concordance across expansions. In our experience with NIGMS repository samples that have more pronounced and clearly defined disease causing CNVs (from cytogenetics and array copy number analyses performed in the course of routine sample quality control) we note that they are generally stable across LCL expansions and fall into a different category of mutation than what has been addressed in the current study.

Taken together, these results are most applicable to genome-wide association studies (GWAS). By design, genome-wide association studies gain statistical power to detect modest disease risk effect sizes with increased sample sizes. One way in which GWAS can increase sample size is to leverage existing clinical samples and biorepositories that offer DNA extracted from LCLs. Our results support this strategy and suggest that SNPs can be reliably measured from LCL DNA. GWAS typically include data from over one million directly measured SNPs, and often include SNPs that are indirectly inferred via imputation. Our documented SNP concordance ranges (99.90–99.98%) across cell line expansions are comparable to reported success rates for SNPs assayed with the Affymetrix 6.0 genome-wide SNP array (99.9%) [9], and higher than reported success rates for imputed GWAS SNPs (< 99.8% [15, 16]). Our results therefore support the use of LCL DNA as an important resource for GWAS. More caution should be taken in studies that are focused on the accurate measurement of germline structural variation in culture given the documented changes in CNV loci across LCL transformation, passaging, and expansion.

## Limitations

The primary limitation of the current study is the sample size of six research subjects. In addition, CNV calls across technical replicates using the Affymetrix SNP 6.0 array data were also inconsistent and suggestive that data collection more focused on CNV calls such as array CGH may be needed to investigate CNV stability in culture.

## Additional files

**Additional file 1: Figure S1.** Diagram of sample generation. For each of the six research participants, six tubes of whole blood were collected. DNA was extracted from one tube of whole blood, and the remaining five tubes were transformed into lymphoblastoid cell lines (LCLs). Each LCL was expanded three times. DNA was extracted from each LCL and each expansion for analysis.

**Additional file 2: Table S1.** The list of samples that were included in the study.

**Additional file 3: Table S2.** The list of copy number variants that were identified in the study.

## Abbreviations

LCL: lymphoblastoid cell line
SNP: single nucleotide polymorphism
CNV: copy number variants
PBMC: peripheral blood mononuclear cells
