# Supplementary figures and images for: Genetic and genomic stability across lymphoblastoid cell line expansions
Source: BMC Res Notes. 2018 Aug 3;11:558. doi: 10.1186/s13104-018-3664-3 (PMC6076395; doi:10.1186/s13104-018-3664-3)

DNA Source

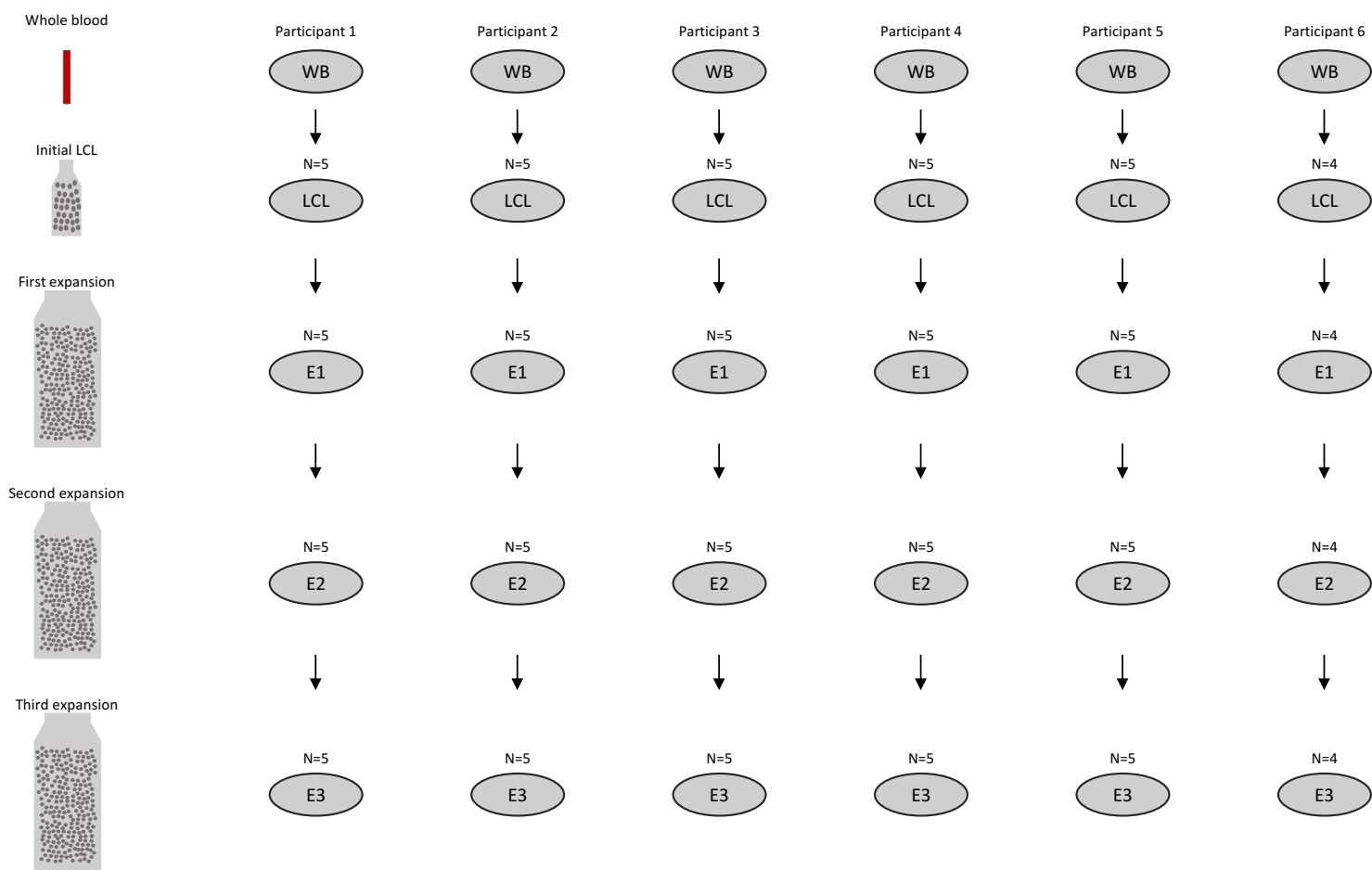

Supplement: Supplementary file 1 — Additional file 1: Figure S1. Diagram of sample generation. For each of the six research participants, six tubes of whole blood were collected. DNA was extracted from one tube of whole blood, and the remaining five tubes were transformed into lymphoblastoid cell lines (LCLs). Each LCL was expanded three times. DNA was extracted from each LCL and each expansion for analysis. [file 13104_2018_3664_MOESM1_ESM.pdf]
