# Supplementary material for: Genetic and genomic stability across lymphoblastoid cell line expansions
Source: BMC Res Notes. 2018 Aug 3;11:558. doi: 10.1186/s13104-018-3664-3 (PMC6076395; doi:10.1186/s13104-018-3664-3)
Supplement: Supplementary file 2 — Additional file 2: Table S1. The list of samples that were included in the study. [file 13104_2018_3664_MOESM2_ESM.pdf]

Subject	Gender	Sample	# Technical Replicates (Expansion)	Cell Type
1	Male	GM22640	2	Buffy Coat
		GM22641	1	B-Lymphocyte
		GM22642	0	B-Lymphocyte
		GM22643	0	B-Lymphocyte
		GM22644	0	B-Lymphocyte
		GM22645	0	B-Lymphocyte
2	Male	GM22646	0	Buffy Coat
		GM22647	1	B-Lymphocyte
		GM22648	0	B-Lymphocyte
		GM22649	0	B-Lymphocyte
		GM22650	0	B-Lymphocyte
		GM22651	0	B-Lymphocyte
3	Female	GM22670	0	Buffy Coat
		GM22671	0	B-Lymphocyte
		GM22672	0	B-Lymphocyte
		GM22673	1	B-Lymphocyte
		GM22674	0	B-Lymphocyte
		GM22675	0	B-Lymphocyte
4	Female	GM22676	0	Buffy Coat
		GM22677	0	B-Lymphocyte
		GM22678	0	B-Lymphocyte
		GM22679	0	B-Lymphocyte
		GM22680	0	B-Lymphocyte
		GM22681	0	B-Lymphocyte
5	Male	GM22730	1	Buffy Coat
		GM22731	0	B-Lymphocyte
		GM22732	1	B-Lymphocyte
		GM22733	0	B-Lymphocyte
		GM22734	0	B-Lymphocyte
		GM22735	0	B-Lymphocyte
6	Female	GM22736	0	Buffy Coat
		GM22737	0	B-Lymphocyte
		GM22738	0	B-Lymphocyte
		GM22739	0	B-Lymphocyte
		GM22741	0	B-Lymphocyte
