# Supplementary material for: Genetic and genomic stability across lymphoblastoid cell line expansions
Source: BMC Res Notes. 2018 Aug 3;11:558. doi: 10.1186/s13104-018-3664-3 (PMC6076395; doi:10.1186/s13104-018-3664-3)
Supplement: Supplementary file 3 [file 13104_2018_3664_MOESM3_ESM.docx]

chr:start-end (HG37)	Copy Number	Individual	immunoglobulin region
chr14:106973708-107167604	1	1	yes
chr14:22612288-22978577	1	1	yes
chr17:29395746-80244067	3	1	
chr17:43994648-44157597	1	1	
chr17:44187492-44280697	1	1	
chr22:22719864-23222208	1	1	yes
chr7:142342355-142493129	1	1	
chr7:38325778-38377925	1	1	
chr1:200960886-201029431	1	2	
chr14:106715489-107216306	1	2	yes
chr14:22481451-22978775	1	2	yes
chr5:103789492-104869290	3	2	
chr10:47085303--47703613	3	3	
chr14:106973708-107145272	1	3	yes
chr14:22545787-22977249	1	3	yes
chr16:68403966-68530889	1	3	
chr2:37978120-38023225	1	3	
chr6:25840485-26318903	3	3	
chr11:114841606-115368517	3	4	
chr14:22450091-22988571	1	4	yes
chr22:22726670-23078808	1	4	yes
chr7:135675380-135813196	1	4	
chr7:38303120-38398047	1	4	
chr11:25136547-25293551	1	5	
chr14:22613690-22977249	1	5	yes
chr2:194854188-195053481	0	5	
chr22:22719864-23112535	0	5	yes
chr7:38303120-38386791	1	5	
chr13:38071135-38128643	1	6	
chr14:22478961-22883939	1	6	
chr22:22386434-23112535	1	6	yes
chr22:35555524-35658022	1	6	
chr3:97068564-97286061	0	6	
chr7:142126531-142213677	1	6	
chr7:38303120-38386791	1	6	
